# Patient’s Perception of the Role of Gym Activity in Abdominal Wall Herniation in Adults: A Prospective Study

**DOI:** 10.1186/s40798-024-00749-x

**Published:** 2024-08-13

**Authors:** Prabir Boruah, Rabbani Mahmoud ElSayed Hassan Daoud, Dylan Viani Walsh, Natallia Kharytaniuk, Salim Fredericks, James Ryan, Asila Abdelatif, Nuha Birido, Thomas Noel Walsh

**Affiliations:** 1 Department of Surgery, Royal College of Surgeons in Ireland, Connolly Hospital, Dublin, Ireland; 2 Royal College of Surgeons in Ireland - Medical University Bahrain, Busaiteen, Bahrain; 3 Academic Department of Surgery, Connolly Hospital, Blanchardstown, Dublin, Ireland; 4 Department of Surgery, Royal College of Surgeons in Ireland – Medical University Bahrain, Busaiteen, Bahrain; 5 Department of Biochemistry, Royal College of Surgeons in Ireland – Medical University Bahrain, Busaiteen, Bahrain

**Keywords:** Hernia, Gym, Lifting, Aetiology, Pilates, Patient Awareness, Work-practice Changes, Prospective Study

## Abstract

**Background:**

Despite significant changes in healthcare, work practices, and leisure activity, the proposed precipitating factors for abdominal wall hernias have remained largely unchanged for almost two centuries. We aimed to investigate if there have been shifts in these factors over time by examining patients’ perception of precipitating factors for abdominal wall hernia development. This study was conducted in the Royal College of Surgeons In Ireland Department of Surgery, Connolly Hospital, Blanchardstown, Dublin, where patients with abdominal wall hernias completed a questionnaire  .

**Results:**

A total of 277 patients (mean age 55.7; 85.6% male) with abdominal wall hernia completed the questionnaire (66.1% inguinal; 10.8% umbilical; 6.9% paraumbilical; 10.5% epigastric; 3.2% incisional; 1.4% femoral, and 1.1% port-site). One hundred and twenty patients (43.3%) believed their hernia was due to lifting, 71 (25.6%) cited gym activity and 17 (6.1%) cited other sporting activities as the precipitating factor. Traditional factors – chronic cough and constipation - were cumulatively cited only by 11 patients (4.0%), while prostatic obstruction was not cited by any.

**Conclusion:**

This study suggests that fitness pursuits may be an increasing contributor to the development of abdominal wall hernia. Greater attention should be paid to the proper use of gym equipment to minimise the risk of hernia development.

## Key findings

Almost one quarter of patients attributed their hernia to gym activity which should be further explored in prospective studies.

This potentially reflects changes in work practice and lifestyle.

Traditional causes like chronic cough and constipation were rarely cited, probably reflecting improved healthcare and lifestyle practices.

## Background

The true incidence of body wall hernia is unclear but older studies have suggested that up to 27% of males and 3% of females will develop a hernia during their lifetime [1] and a further 20% of males have a patent processus vaginalis at autopsy [2, 3]. Not only are abdominal wall hernias more common in men, but they are also age-related, with the incidence of male hernias rising from 7.3% at ages 20–39, to 22.8% at ages 60–74 [4].

The medical literature suggests that a range of factors may predispose to abdominal wall herniation. The most evidence-based of these include connective tissue disorders (5,6), abnormal fascial hydroxyproline [5–7] defective collagen type III [6, 8, 9], malnutrition [10], smoking [11], patent processus vaginalis [12] and family history [13]; all of which have been shown to contribute to abdominal wall weakness. Indeed, some have insisted that these factors are so pernicious as to make hernia almost “inevitable” in these subjects [14].

Despite the presence of predisposing factors, a hernia is unlikely to develop without a precipitating factor, which invariably contributes to hernia through the elevation of intra-abdominal pressure. The precipitating factors most frequently cited in textbooks include lifting, chronic cough, chronic constipation, prostatic outflow obstruction, and obesity [15–17]. These have remained unchanged for almost two centuries, since first proposed by Astley Cooper in 1844 [18], despite changes in population health, work practices, and leisure pursuits. Machines have largely replaced humans for heavy lifting [19–21]. Family medicine has improved dramatically resulting in earlier, and more efficient, medical interventions for intestinal, prostatic, and pulmonary risk factors [22, 23]. While obesity has been historically associated with hernia development, recent literature suggests that high BMI actually exerts a protective effect against hernia development [24–26]. Respiratory health has improved as a result of a reduction in smoking [27–29] and smoke-free work and leisure environments [30]. Leisure activity has also changed with a “fitness revolution” leading to an explosion in gym use and increased exercise and fitness activities [31].

An unpublished pilot study, performed at our unit, found that many patients mentioned gym activity as a probable cause of their hernia, which prompted further exploration through prospective data collection. We hypothesise that changes in work practices, health, and leisure pursuits may be reflected in a change in risk-factors for abdominal wall hernia. The aim of this study is to report the risk-factors that a consecutive series of patients perceived as having precipitated their hernia, taking note of the particular settings and activities in which the patients were engaging prior to developing their hernia.

## Methods

### Study Outline

This prospective study was conducted in the Royal College of Surgeons In Ireland Department of Surgery, Connolly Hospital, Blanchardstown, Dublin, over an 18 months period from March 2017 to August 2018. Consecutive patients presenting with an abdominal wall hernia to a single surgical unit were studied.

### Study Design

**Fig. 1:**
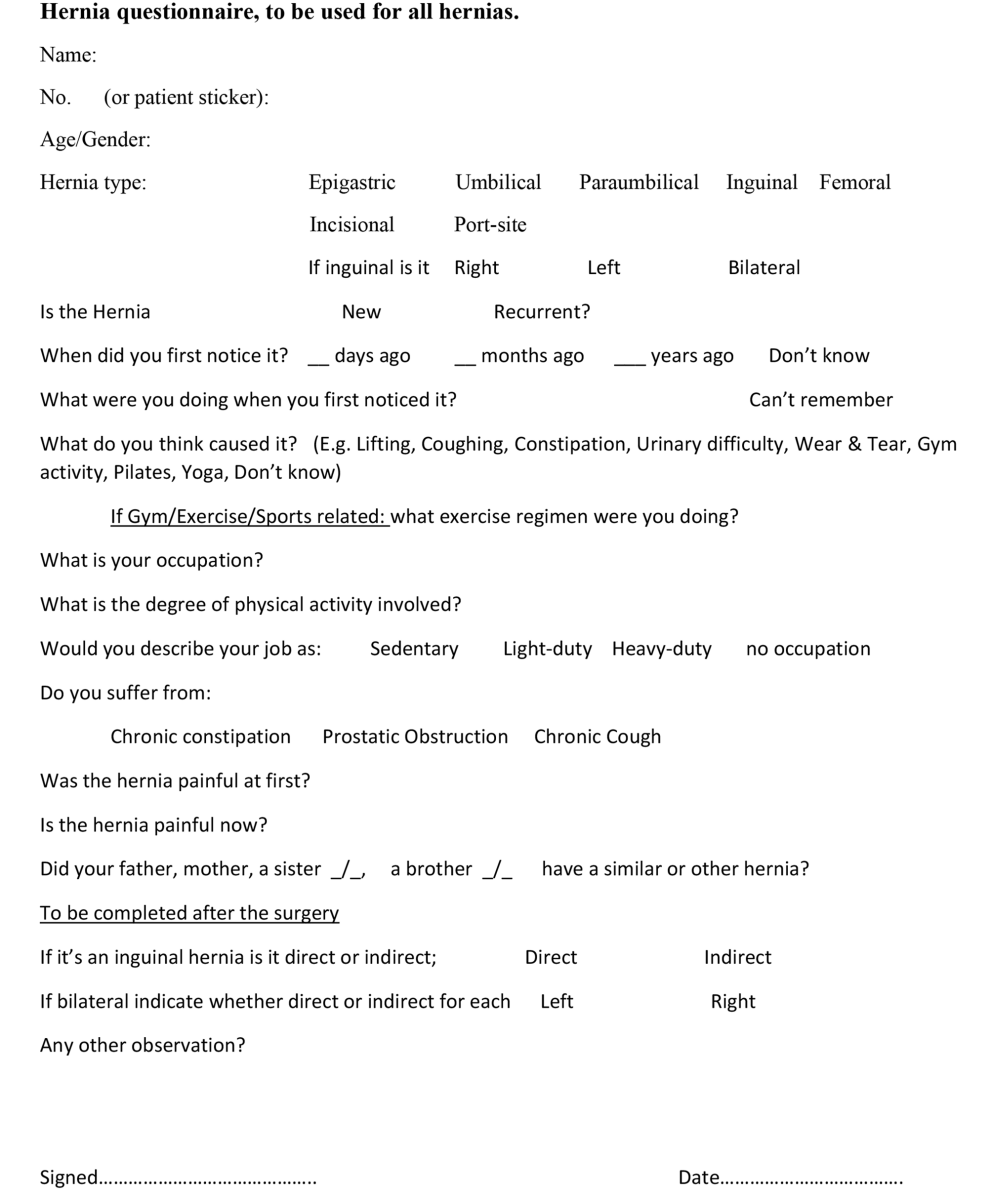
Hernia questionnaire

All patients referred with an abdominal wall hernia to a single surgical unit underwent a detailed history and clinical examination. Once a hernia diagnosis was confirmed, they were presented with a questionnaire focusing on what they believed was the cause of their hernia **(**Fig. 1**)**. The questionnaire was completed by a doctor who asked all of the relevant questions and if necessary paraphrased the questions for better understanding, and clarified the patients answers. The answers were recorded on the questionnaire, which was included in the patients notes and later transferred to an Excel database. Following surgery, the type of hernia was confirmed, and whether direct or indirect if an inguinal hernia.

### Inclusion/Exclusion Criteria

All participants were over 18 years of age and all gave verbal consent to its completion. Exclusion criteria were age under 18 and patients with cognitive impairment.

### Data Collection

The admitting doctor asked the patient hernia-related questions from the study questionnaire which was completed, detailing the operative findings, prior to discharge from the hospital. The information gathered focused on the type and location of the hernia, whether new or recurrent, when first identified and whether there was a family hernia history. Patients were specifically asked what activity they believed contributed to the development of their hernia and a list of prompts were provided including “lifting”, “gym activity”, and “don’t know”. The classical risk factors were specifically enquired about, such as a chronic cough, constipation and prostatic outlet obstruction. Patients were questioned on the degree of physical activity of their occupations which were categorised into sedentary, light- and heavy-duty occupations. Sedentary occupations included office work such as secretarial and information technology (IT) workers; light duty occupations included grocery shopkeepers and schoolteachers; and occupations such as construction workers were considered heavy duty (Table 1). Some answers were gathered by encircling items from the menu of responses (coughing, constipation etc.), and some were collected in free-text (e.g. occupation). Weightlifting in the gym was categorised under gym activity to separate it from occupational reasons for heavy lifting.

**Table 1:** Demographics in relation to hernia type

Types		N (%)	Mean Age	SD
Inguinal	All	183 (66.1)	58	15.3 ^†^
	Male	175 (95.6%)	58	15.3 ^†^
	Female	8 (4.4%)	64	14.0 ^†^
Umbilical	All	30 (10.8%)	51	13.6
	Male	24 (80.0%)	54	13.6
	Female	6 (20.0%)	42	10.0
Epigastric	All	29 (10.5%)	44	12.2 ^‡^
	Male	18 (62.1%)	46	11.4
	Female	11 (37.9%)	42	13.5
Paraumbilical	All	19 (6.9%)	50	13.4
	Male	14 (73.7%)	54	13.0
	Female	5 (26.3%)	39	7.4
Incisional	All	9 (3.2%)	62	16.7
	Male	5 (55.6%)	63	19.9
	Female	4 (44.4%)	61	14.2
Femoral	All	4 (1.4%)	59	18.6
	Male	0 (0.0%)	N/A	N/A
	Female	4 (100.0%)	59	18.6
Port-site	All	3 (1.1%)	54	15.3
	Male	1 (33.3%)	67	N/A
	Female	2 (66.7%)	47	14.1
Total	All	277 (100.0%)	56	15.4
	Male	237 (85.6%)	57	15.1
	Female	40 (14.4%)	50	15.7

ANOVA showed a significant difference in age across hernia types for all, male and female

† Significant difference in age between epigastric and inguinal

‡ Significant difference in age between epigastric incisional

### Data Analysis

All data were collected on a paper questionnaire and transferred to an anonymised Excel database. Descriptive statistics were used to summarise categorical characteristics and demographic data of patients. Continuous variables were reported as mean (standard deviation) where appropriate. Categorical variables were presented as proportions and percentages. Continuous variables were compared using an independent Student’s t-test or Mann-Whitney U test, were appropriate. Chi-squared test or Fisher’s exact test was used to compare categorical data. ANOVA was used to compare mean ages across groups. Post hoc tests were used to compare ages for the hernia types. All statistical analyses were performed using IBM SPSS Statistics software version 28.0 for Windows IBM Corp, Chicago, IL. Statistical significance was considered to be *p* < 0.05 (two-sided).

## Results

### Patient Demographics

Two hundred seventyseven patients with abdominal wall hernia that were seen at our surgical unit between March 2017 and August 2018 completed the questionnaire. The mean (range) age in years of the entire cohort was 55.7 (18 to 90), and 237 (85.6%) were male. There was a significant difference in mean (s.d.) age between male [57 (15.1)] years and female [50 (15)] patients (*p* = 0.01) (Table 1).

### Hernia Type, Age and Gender Distribution

Of 277 patients with body wall hernias, 183 (66.1%) were inguinal, of which 95.6% were in males, 101 (55.2%) were right sided, 61 (33.3%) were left sided, 19 (10.4%) were bilateral, and 2 (1.1%) were not recorded. Umbilical and paraumbilical hernias accounted for 30 (10.8%) and 19 (6.9%) patients, respectively, of which 38 (77.6%) were males. There were 29 (10.5%) epigastric hernias of which 62.1% were in males (Table 1). There were 9 (3.2%) incisional hernias, 5 (55.6%) of which were in males. Only 4 (1.4%) femoral hernias were reported, and all occurred in females. There were 3 (1.1%) port-site hernias, of which 2 (66.7%) were in males. Two hundred and forty-one (87%) of the hernias were new and 32 (11.6%) were recurrent, and in 4 (1.4%) this information was missing.

When types of hernia were compared there was a significant difference in mean ages across the different types of hernia groups (one-way ANOVA *p* < 0.001). Patients with an epigastric hernia were younger than incisional and inguinal groups (*p* < 0.05) based on post hoc tests. The age distribution across different hernia groups is summarised in Table 1.

### Family History

Seventy-five (27.1%) patients reported a family history of hernia. The mean (s.d.) age of patients with a family history at the time of diagnosis was 52 (14.7) years, which was significantly younger than those without a family history of hernia who were 57 (15.5) years old (*p* = 0.03).

### Patient-reported Causes of Hernia

Heavy lifting was the most frequently reported precipitating cause for abdominal wall hernia cited by 120 (43.3%) patients. Gym activity was the second most frequently reported cause, cited by 71 (25.6%) patients. Other sporting activities were cited by 17 patients (6.1%), and 21 patients (7.6%) cited “wear-and-tear”. Pregnancy was cited by 8 (2.9% of all patients; 20% of females) female patients, and 8 (2.9%) patients cited other specific causes such as household chores or getting out of a car. Finally, 21 patients (7.6%) could not attribute their hernia to a specific cause.

Of the classically cited causes, cough was cited by only 10 (3.6%) patients, only 1 (0.4%) patient cited constipation and no patient cited prostatic obstruction. When specifically asked about these symptoms, 24 (8.6%) patients reported that they suffered from constipation, 12 (4.3%) reported chronic cough and 8 (2.9%) admitted to prostatic obstruction. These patients, however, did not always attribute their hernia to these symptoms. Of the 24 patients with constipation, 12 (50%) attributed their hernia to lifting, 5 didn’t know, 4 attributed it to gym activity, 2 to wear and tear and 1 to coughing. Again, of the 12 patients with a chronic cough, 6 attributed their hernia to lifting, 4 to coughing and 2 to gym activity. Finally, of the 8 patients that admitted to prostatic obstruction, 6 attributed their hernia to lifting and 2 to gym activity.

### Initial Recognition of the Hernia by the Patient

One hundred thirty-one (47.3%) patients first noticed their hernia while engaging in daily-life activities such as walking, carrying, showering getting into car etc. Seventy-seven (27.8%) patients could not recall the first time they noticed their hernia. Twenty-three patients (8.3%) first noticed their hernia in a gym facility but only 32.4% of gym-related hernias were first noticed while in the gym. Among the rest, 13 saw or felt something at the hernia site, 7 had their hernia identified by a doctor, 4 noticed it while coughing, 4 while in the toilet and 4 had their attention drawn to it because of pregnancy.

### Relationship between cause of Hernia and Lifestyle/Work Grade

Patient’s level of activity was defined as sedentary in 49.1%, light-duty in 23.6% and heavy-duty in 27.3% (Table 2**)**. Two hundred and sixty seven were included as 10 patients had no occupation. Of the group that cited gym activity as the most-likely cause of their hernia, a significantly higher proportion had a sedentary occupation 42 (63.6%), compared to 15 (22.7%) who had a light-duty occupation and 9 (1.73%) with a heavy-duty occupation (chi-squared = 9.9, d.f. = 2, *p* = 0.01). In contrast a significantly higher proportion of patients that attributed their hernia to lifting had heavy-duty jobs, as opposed to light-duty or sedentary occupations (chi-squared = 46.5, d.f. = 2, *p* < 0.001). Patients were twice as likely to attribute their hernia to gym activity if they had a sedentary occupation compared to a heavy-duty job [odd ratio: 2.4 (1.3–4.4)]. Conversely, patients were nine times more likely to attribute their hernia to lifting activity if they did a heavy-duty job rather than a sedentary one [odd ratio: 8.7 (4.5–16.9)].

**Table 2:** Probable causes of hernia in relation to patients’ occupation. Frequencies were compared across the three job-categories using the Chi-squared test. * 10 patients were excluded because they cited “no occupation”

Cause	(*N* = 267)*	Sedentary (*N* = 131)	Light-duty (*N* = 63)	Heavy-duty (*N* = 73)	*P*-value
Lifting	118 (44.2%)	36 (30.5%)	26 (22.0%)	56 (47.5%)	< 0.001 ^†^
Gym activity	66 (24.7%)	42 (63.6%)	15 (22.7%)	9 (13.7%)	0.007 ^‡^
Wear & tear	21 (7.9%)	13 (61.9%)	7 (33.3%)	1 (4.8%)	0.053
Don’t know	20 (7.5%)	15 (75.0%)	2 (10.0%)	3 (15.0%)	0.051
Sporting	17 (6.4%)	10 (58.8%)	4 (23.5%)	3 (17.7%)	0.612
Cough	10 (3.7%)	4 (40.0%)	5 (50.0%)	1 (10.0%)	0.118
Pregnancy	8 (3.0%)	7 (87.5%)	1 (12.5%)	0 (0.0%)	0.060
Other	7 (2.6%)	4 (57.1%)	3 (42.9%)	0 (0.0%)	0.208

NS: no significant difference

† - Patients were nine times more likely to attribute their hernia to lifting activity if they carried out a heavy-duty job rather than a sedentary occupation [odd ratio : 8.7 (4.5–16.9)]

‡ - Patients were twice as likely to attribute their hernia to gym activity if they had a sedentary occupation compared to a heavy-duty job [odd ratio : 2.4 (1.3–4.4)]

No other significant differences were seen between the sedentary and heavy-duty occupation categories for the other possible causes. No significant differences were found for categories: wear and tear, don’t know, sporting, cough, pregnancy. The distribution of job level of physical activity across different hernia types is illustrated in Table 2.

### Sub-analysis of the Patients Attributing Their Hernia to gym Activity

Seventy-one patients attributed their hernia to exercise activity in the gym. This group was compared with the 206 other patients attributing their hernia to other causes (Table 3). No statistically significant differences were identified between these groups (non-gym and gym activity) regarding frequencies related to sex, instance, family history, side, and type of hernia.

**Table 3:** Comparison of hernia frequencies for those who patient attributing their hernia to gym activity and those who attributed it to other causes

Category	variables	Gym-related hernia group	Non Gym-related hernia group	*p*-value
Sex	Male	59	178	NS
	Female	11	29	
Instance	New	65	176	NS
	Recurrent	5	27	
Family History	Yes	25	50	NS
	No	44	153	
Side	Right	26	78	NS
	Left	17	46	
	Bilateral	3	16	
	Mid	0	4	
	Not recorded	24	63	
Type	Epigastric	13	16	NS
	Inguinal	45	138	
	Incisional	1	8	
	Paraumbilical	6	13	
	Umbilical	5	25	
	Femoral	0	4	
	Port-site	0	3	

## Discussion

Our study found that gym activity came second only to lifting on the list of precipitating factors for abdominal wall hernia, as perceived by our patients. To the best of our knowledge, this is the first report to identify gym activity as a major precipitating factor for abdominal wall hernia. This probably reflects the “fitness revolution” which has swept Europe and North America over the past twenty years [31]. Over 21% of the Swedish population, 17% of the Dutch population and 10% of the Irish population have gym membership while probably as many more pursue fitness goals through sports clubs and small independent, and other facilities [32]. Only 36% of patients who believed that their hernia was gym related were in the gym when they discovered it - but this is consistent with the finding that over 20% of patients are unaware of the presence of a hernia at the time of an emergency presentation [33]. It is possible, however, that when specifically asked which factors were likely to have caused the hernia gym-users may have been prompted by “gym activity” being available on the list of options.

The traditionally cited precipitating factors, such as coughing, constipation and urinary outflow obstruction, were cumulatively cited by only 4% of patients. Even when specifically asked, only 15.8% of hernia patients admitted to having these symptoms. These figures are hardly surprising when we consider the changes in health care in the two centuries since Astley Cooper’s 1844 observations [18]. Reduction in smoking rates [27, 29], improved environmental air and improvement in respiratory health has reduced the incidence of chronic cough, especially in males [34, 35]. And increased access to family doctors and specialists have reduced the incidence of untreated chronic constipation or prostatic outlet obstruction [22]. However, in resource limited countries, these causes remain prevalent. Agarwal et al. examined the causes of hernia in a population from Northern India and reported that altered bowel habits (mainly constipation) was the second most common cause of hernia (36.36%) following lifting (55%). In addition, 40% of their patients had respiratory problems compared to our study where only 12 (4.3%) complained of chronic cough [36]. Obesity, also cited in textbooks [18] has been proven to be protective for herniation in a recent studies [24–26].

Heavy lifting, whether or not work related, remained the most frequently cited probable contributor to abdominal wall hernia in our patients population (43.3%). Kang et al. found in a retrospective analysis of over thirty thousand patients that labourers, machine operators, and mechanics had the highest incidence of hernia [37]. A meta-analysis by Kuijer et al. supported the association in male workers between lateral hernias and standing and walking for > 6 h each working day, or lifting more than 4,000 kg per workday [38]. Vad et al. made a very persuasive case that work-related lifting and standing contributed to the development of a hernia by shortening the rate advancement period (RAP). They concluded that reducing the time spent on walking and standing could potentially prevent 30% of inguinal hernias [39]. Work or leisure related lifting and straining manoeuvres increases intra-abdominal pressure (IAP) [40] and an association between lifting and hernia has long been recognized [41, 42]. IAP is thought to force preperitoneal fat through the attenuated strands of the linea alba or through the inguinal canal where such fatty protrusions could act as the “leading edge” for herniation [43, 44].

It is not surprising that the incidence of inguinal hernia repair has reduced over the past several decades. The incidence of inguinal hernia repair reduced from 217 to 194 [45], 240.8 to 217.1 [46], and 474 to 373 [47] per 100,000 population over a period ranging from 15 to 43 years in Australia, the UK, and the USA respectively. Most occupations today are sedentary or semi-sedentary [48] and even where lifting is required, lifting equipment and courses on manual handling have been introduced to avoid musculoskeletal injuries [21, 49, 50]. Feedback from industry supports the view that safe lifting practice reduces work related injuries [51–53]. Our findings become relevant to people with these occupations as 63.6% of our patients who attributed their hernia to gym activity had a sedentary occupation, and an additional 22.7% had only light-lifting duties.

While no previous publication has linked gym activity to abdominal hernia, it would be prudent of the fitness community to be aware of this risk. In the clinical sphere, litigation for hernia repair is increasing in the UK [54] and in the US [55]. There were more than 19,000 hernia mesh lawsuits as of June 2023 [56] resulting in 5.3 million USD being awarded for laparoscopic hernia repair claimants and 12.1 million USD for claimant of open hernia repairs [57], paralleling the bourgeoning class action lawsuits against mesh companies for post-repair inguinodynia [58]. Many industries have modified their work practices to address work related disorders and avoid litigation [59]. In the healthcare sector, ergonomic interventions were introduced to minimize work-related musculoskeletal disorders among nurses and improve the working environment in hospitals [60]. Gym proprietors would be wise to proactively address the risk of litigation by encouraging stricter adherence to guidelines on the use of gym equipment as it is likely just a matter of time before they are indited for inadequate tuition or supervision on equipment usage. Suggested initiatives by gym proprietors could include displaying locker room posters about the possibility of hernia development, how to recognize it, and most importantly how to avoid it. Some exercise related websites have already started providing advice on how to avoid abdominal wall hernia through better lifting techniques [61]. Additionally, it would be wise to document a history of predisposition towards abdominal wall hernia, especially a family history of hernia and a history of a surgical incision. Subjects with a recent abdominal incision, particularly through the *linea alba*, may be advised to see the gym doctor, even though there is no consensus on what, if any, and for how long, restriction to lifting and straining activity should be observed after abdominal wall surgery [62]. Measures should be considered to establish the degree of physical activity to which the gym user may safely be exposed [63]. This would also provide evidence of gym compliance with standards, in the event of legal action. Gym owners may also choose to create disclaimers to protect against potential litigations.

The study’s strength lies in the fact that it was conducted prospectively when the patient first presented to the clinic, and often not knowing what the swelling was. This information was also given before the outcome of the repair was known or before any chronic postoperative pain might have biased their recollection. Our study is subject to potential biases and confounding factors. Selection bias may arise from the study’s focus on a single surgical unit located in an urban area. Recall bias is also a concern as many could not recall what they were doing when they identified their hernia, and only 36% of gym-related hernias were first noticed while in the gym. Patient-reported “cause of hernia” is also prone to bias as most non-medical individuals have a poor understanding of abdominal wall anatomy and hernia pathophysiology [64]. Patients may have been prompted to choose gym as it was an available option and not because they initially attributed it to the hernia. Unfortunately we were not able to compare different types of gym activities with hernia formation in this study, but future studies are encouraged to explore this further. It is unclear from our data whether subjects who developed herniation were abiding by gym guidelines, but the counter argument is that not all gyms provide adequate advice on the use of gym equipment.

## Conclusion

In conclusion, we found that the second most frequently cited cause of herniation among our sample pool of patients was gym activity, and traditional risk factors such as chronic cough, constipation and prostatic obstruction were relatively rare. We do not suggest that people desist from gym activity: instead, we would encourage greater compliance with guidelines on the use of gym equipment and exercise regimens. Gym owners and fitness instructors should consider the potential for litigation and introduce safety measures, such as insisting on closer attention to guidelines when using equipment and exercise regimens. A prospective longitudinal image guided study of body wall integrity of applicants signing up for gym membership, would be an important first step in the study of an association between gym-related activity and the development of abdominal wall hernia.

## Data Availability

The data that support the findings of this study are not openly available due to reasons of sensitivity and are available from the corresponding author upon reasonable request.

## Abbreviations

ANOVA: Analysis of variance
SD: Standard deviation
IAP: Intra-abdominal pressure
RAP: Rate advancement period
